# Risk factors for thromboembolism during first-line treatment of patients with unresectable advanced or recurrent colorectal cancer: a retrospective short study

**DOI:** 10.1186/s40780-023-00291-0

**Published:** 2023-07-03

**Authors:** Ryo Takada, Miki Fujiwara, Masatoshi Maki, Yoko Takahashi, Koji Tamura

**Affiliations:** Department of Hospital Pharmacy, National Hospital Organization Fukuyama Medical Center, 4-14-17 Okinogami-cho, 720-8520 Fukuyama-city, Hiroshima, Japan

**Keywords:** Colorectal cancer, Epidermal growth factor receptor inhibitors, Thromboembolism, Vascular endothelial growth factor inhibitors

## Abstract

**Background:**

While cancer is a risk factor for developing thromboembolism, so is the use of molecularly targeted therapies. This study aimed to determine whether thromboembolism incidence differed between vascular endothelial growth factor (VEGF) and epidermal growth factor receptor (EGFR) inhibitor use in patients with unresectable advanced or recurrent colorectal cancer, and to compare the risk of thromboembolism caused by cancer and the use of molecular targeted therapy drugs.

**Main body:**

We retrospectively evaluated patients with unresectable advanced or recurrent colorectal cancer who were treated with a cytotoxic anticancer drug and a VEGF or EGFR inhibitor combination between April 2016 and October 2021. Patients were compared in terms of the regimen administered, thromboembolism occurrence during the first-line treatment period, patient background, and clinical laboratory values. Of the 179 included patients, 12 of 134 (8.9%) in the VEGF-inhibitor group and 8 of 45 (17.8%) in the EGFR-inhibitor group developed thromboembolism, with no significant difference between the groups (*P* = 0.11). There was no significant difference in time to thromboembolism between patients in the VEGF- inhibitor group and patients in the EGFR-inhibitor group (*P* = 0.206). The cutoff value determined by a receiver operating characteristic analysis for the occurrence of thromboembolism was one point. Multivariate analysis using the occurrence of thromboembolism as the response variable identified at least one risk factor for thromboembolism (odds ratio = 4.17, *P* = 0.006, 95% confidence interval = 1.51–11.50). Molecular targeted therapies were not identified as a risk factor.

**Conclusions:**

Although the small sample size, there was no difference in the incidence of thromboembolism between the two molecular-targeted therapies in first-line treatment of patients with unresectable advanced or recurrent colorectal cancer. Our results suggest that risk factors for thromboembolism may be more strongly influenced by cancer itself than by the use of molecularly targeted therapies.

## Background

Chemotherapy can improve survival and palliate symptoms of unresectable advanced or recurrent colorectal cancer, and combined administration of a cytotoxic anticancer drug and molecular-targeted therapy, such as vascular endothelial growth factor (VEGF) and epidermal growth factor receptor (EGFR) inhibitors [1]. Hypertension and proteinuria are typical side effects of VEGF inhibitors, and skin disorders are associated with EGFR inhibitor use. Both drugs are associated with thromboembolism, albeit less frequently.

The incidence of thromboembolism is 4–7 times higher in patients with cancer than in those without [2, 3]. Moreover, its annual incidence is 3–5% in patients with colorectal cancer [3], which in itself is a risk factor for thromboembolism development. Regarding drug-related effects, thromboembolism incidence is 11.9% when bevacizumab, a VEGF inhibitor, is used for chemotherapy [4]. Although the effects of EGFR inhibitors, a meta-analysis demonstrated a 1.34-fold increase in thromboembolism incidence in the drug group than that in the control group [5].

However, the incidence of thromboembolism among Japanese patients with colorectal cancer treated with VEGF inhibitors has not been compared with that in patients administered EGFR inhibitors. Additionally, whether cancer itself, and not just the use of molecular-targeted therapies, directly influences the incidence of thromboembolism in patients with colorectal cancer remains unclear.

This study aimed to compare the difference in thromboembolism incidence between molecular-targeted agents in patients with unresectable advanced or recurrent colorectal cancer concurrently treated with a cytotoxic anticancer drug and VEGF or EGFR inhibitor, and to determine whether molecular-targeted drug administration or cancer has a greater impact on thromboembolism development.

## Main text

This was a single-center retrospective cohort study that aimed to determine whether thromboembolism incidence differs between VEGF and EGFR inhibitors administered to patients with unresectable advanced or recurrent colorectal cancer, and whether molecular-targeted therapies increase thromboembolism incidence compared with the cancer-bearing condition. We retrospectively analyzed data of 224 patients with unresectable advanced or recurrent colorectal cancer from the Fukuyama Medical Center for Patients, who were treated with concomitant cytotoxic anticancer drug and VEGF or EGFR inhibitor between April 2016 and October 2021. Patients were included only during first-line colorectal cancer treatment. Patients not concomitantly using molecular-targeted agents, those without computed tomography (CT) scan between initiation and completion of first-line treatment, those taking antiplatelet agents or anticoagulants prior to first-line treatment, or those with missing data were excluded. The following parameters were evaluated: sex; age; body weight; body surface area; body mass index (BMI); primary colorectal cancer site; presence or absence of metastases and metastatic sites; type and number of cytotoxic anticancer drugs and molecular-targeted therapies; number of first-line treatments; presence or absence of central venous (CV) port; concomitant heart disease, hypertension, or diabetes mellitus; concomitant use of antiplatelet or anticoagulant medications; concomitant use of hormonal agents or hematopoietic agents; white blood cell (WBC) counts, platelet counts, hemoglobin levels at the start of treatment; and presence of thromboembolism on CT with date and site of occurrence. In addition, the following risk factors for thromboembolism in cancer patients were assigned one point each and summed: WBC count > 11,000 cells/μL, platelet count ≥ 350,000 cells/μL, hemoglobin level < 10 g/dL, and BMI ≥ 25.3 kg/m^2^ [6, 7]. The Mann–Whitney U test was performed to compare continuous variables between groups and Fisher’s exact probability test for categorical variables. For continuous variables, receiver operating characteristic (ROC) analysis was performed to obtain cutoff values, which were then converted to categorical variables. Multiple logistic regression analysis was performed to examine factors affecting thromboembolism. The occurrence of thromboembolism was the response variable, and VEGF or EGFR inhibitor administration was always included as an explanatory variable. In univariate analysis, event was defined as the onset of thromboembolism or completion of first-line treatment, and time to event occurrence was calculated using the Kaplan–Meier method. The log-rank test was used for comparisons between groups. Statistical significance was set at two-sided *P*-value of < 0.05. Statistical analyses were performed using EZR version 1.54 (Saitama Medical Center, Jichi Medical University, Saitama, Japan).

**Fig. 1:**
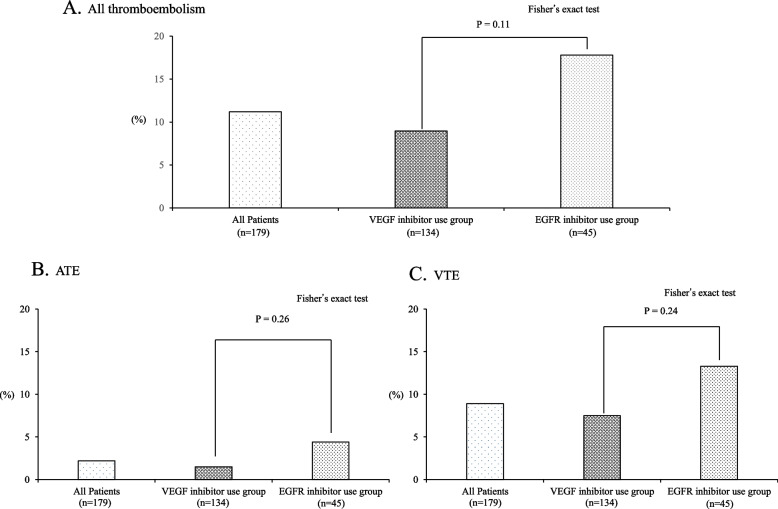
Comparison of thromboembolism incidence between molecular-targeted therapies. Fisher's exact probability test was used for all analyses. The horizontal axis shows the rate of events

**Fig. 2:**
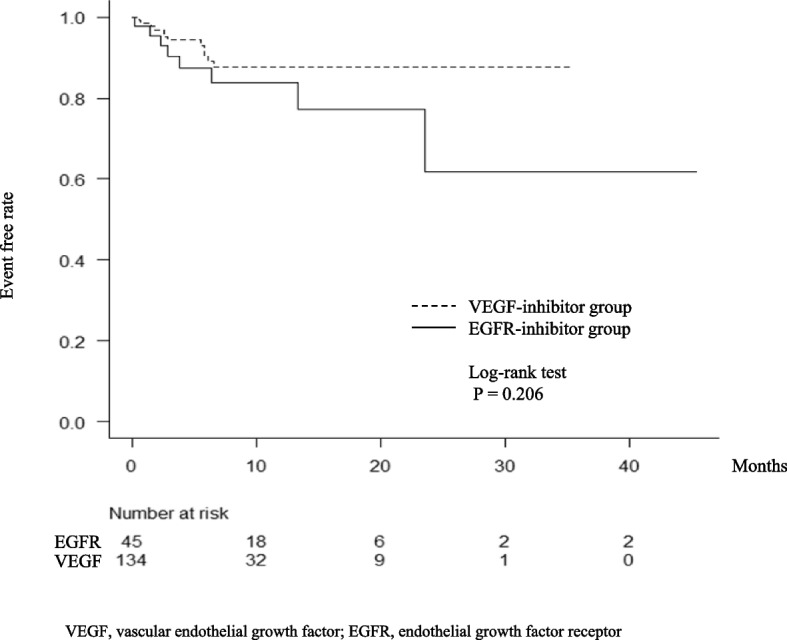
Time to onset thromboembolism incidence between molecular-targeted therapies

Of 179 patients, 134 (74.9%) and 45 (25.1%) were in the VEGF- and EGFR-inhibitor groups, respectively. The percentage of patients with a CV port was significantly higher in the EGFR-inhibitor group than in the VEGF-inhibitor group (*P* < 0.001); however, the other parameters, including the risk factor scores for thromboembolism in cancer patients, did not significantly differ between the groups (Table 1). Thromboembolism incidence was 11.2% (20 patients): 8.9% (8 patients) and 17.8% (12 patients) in the VEGF- and EGFR-inhibitor groups, respectively, showing no significant between-group difference (*P* = 0.11; Fig. 1A). Similarly, no significant differences were observed for arterial thromboembolism (ATE) and venous thromboembolism (VTE) (Figs. 1B and 1C, respectively). The Kaplan–Meier curve for time to thromboembolism onset in the groups was evaluated, and there was no significant difference between the groups (*P* = 0.206; Fig. 2). The cutoff value for the number of risk factors for thromboembolism in cancer patients was one point (Table 2). Univariate analysis using thromboembolism occurrence as the response variable revealed that only one or more risk factors for thromboembolism were extracted as influential factors (*P* = 0.003; Table 3). Similarly, in the multivariate analysis of Model 1 and Model 2, the use of VEGF and EGFR inhibitors was not identified as a risk factor, whereas having at least one risk factor for thromboembolism was identified as a risk factor (*P* = 0.006, *P* = 0.006, respectively; Table 3).

**Table 1:** Patient characteristics

	VEGF-inhibitor group (*n* = 134)	EGFR-inhibitor group (*n* = 45)	*P*-value
Sex (female)	58 (43.3%)	18 (40.0%)	0.731^a)^
Age (year)	69 (33–85)	66 (32–82)	0.345^b)^
Body surface area (m^2^)	1.570 (1.192–2.010)	1.583 (1.223–1.941)	0.717^b)^
BMI (kg/m^2^)	21.9 (15.0–36.5)	21.6 (15.9–33.8)	0.958^b)^
Primary rectal tumor	44 (32.8%)	17 (31.1%)	0.588
Number of metastases (n)	1 (1–5)	1 (1–3)	0.589^b)^
Types of molecular-targeted drugs	Bevacizumab: 134 (100%)	Panitumumab: 40 (88.9%)	-
		Cetuximab: 5 (11.1%)	
Number of cytotoxic anticancer drugs used in combination (3/2/1)	5/120/9	0/45/0	0.075^a)^
Number of first-line treatments (n)	10.5 (1–62)	9 (1–40)	0.454^b)^
CV port construction, yes (n)	110 (82.1%)	45 (100%)	< 0.001^a)^
Comorbid cardiac disease (n)	2 (1.5%)	3 (6.7%)	0.601^a)^
Comorbid hypertension (n)	35 (26.1%)	16 (35.6%)	0.254^a)^
Comorbid diabetes mellitus (n)	17 (12.7%)	5 (11.1%)	1.000^a)^
Concomitant use of hormonal agents (n)	0 (0.0%)	0 (0.0%)	-
Concomitant use of hematopoietic agents (n)	0 (0.0%)	0 (0.0%)	-
WBC count before starting treatment (× 10^3^/μL)	5.55 (2.3–14.4)	5.90 (3.0–11.6)	0.164^b)^
Platelet count before starting treatment (× 10^3^/μL)	230.5 (79–693)	246 (114–454)	0.423^b)^
Hemoglobin level before starting treatment (g/dL)	11.6 (7.9–15.7)	11.6 (6.5–16.4)	0.552^b)^
Number of risk factors (0/1/2/3)	84/41/7/2	25/15/5/0	0.438^a)^
Median number of days from treatment start date to thromboembolism onset date	84.5 (18–200)	101 (7–707)	0.678^b)^

Data are presented as number of cases (%) or median (min–max) values

^a)^ Fisher’s exact test, ^b)^ Mann–Whitney U test

*VEGF* Vascular endothelial growth factor, *EGFR* Endothelial growth factor receptor, *CV* Central venous, *BMI* Body mass index, *WBC* White blood cell

**Table 2:** Cutoff values for the occurrence of thromboembolism for each risk factor in this study

	Cutoff value	AUC	95%CI	Sensitivity	Specificity
BMI (kg/m^2^)	22.9	0.644	0.515–0.773	0.650	0.642
White blood cell count (× 10^3^/μL)	5.0	0.584	0.454–0.715	0.850	0.403
Platelet count (× 10^3^/μL)	219.0	0.581	0.452–0.710	0.750	0.459
Hemoglobin level (g/dL)	15.2	0.545	0.383–0.706	0.200	0.969
number of risk factor (n)	1	0.666	0.554–0.775	0.648	0.700

*BMI* Body mass index, 95% CI, 95% confidence interval, *AUC* Area under the curve

**Table 3:** Factors influencing the occurrence of thromboembolism

	Number of patients	Occurrence of thromboembolism, yes	Univariate analysis			Multivariate analysis					
						Model 1			Model 2		
			OR	95% CI	*P*-value^a)^	OR	95% CI	*P*-value	OR	95% CI	*P*-value
Sex (female)	76	9	1.12	0.39–3.17	0.815						
Age (≧65 years)	110	15	2.01	0.65–7.44	0.228						
Primary lesion (Rectum)	61	7	1.05	0.33–3.02	1.000						
Metastasis in other organs, yes	131	17	2.23	0.60–12.44	0.287						
Comorbid cardiac disease, yes	5	2	5.68	0.45–53.13	0.096						
CV port expansion, yes	155	20	inf	0.81–inf	0.080						
Use of VEGF inhibitors, yes	134	12	0.46	0.16–1.39	0.110	0.48	0.18–1.31	0.153			
Use of EGFR inhibitors, yes	45	8	2.19	0.72–6.34	0.110				2.07	0.76–5.60	0.153
Risk factors for occurrence of thromboembolism≧1	70	14	4.26	1.44–14.30	0.003	4.17	1.51–11.50	0.006	4.17	1.51–11.50	0.006

^a)^Fisher’s exact test

*OR* Odds ratio, 95% CI, 95% confidence interval, *Inf* Infinite, *CV* Central venous, *VEGF* Vascular endothelial growth factor, *EGFR* Endothelial growth factor receptor

Systemic VEGF inhibition by VEGF inhibitors increases systemic vascular events due to decreased production of nitric oxide (NO), which has vasodilating effects [8] and increased resistance of the vascular endothelium [9]. In addition, EGFR inhibitors suppress VEGF production in tumor cells [10], which results in apoptosis of vascular endothelial cells. Inhibition of VEGF production may indirectly inhibit NO production, thereby disrupting the regenerative capacity of vascular endothelial cells, causing vessel wall defects, and leading to thrombosis [11–17]. Altogether, both VEGF and EGFR inhibitors are risk factors for thromboembolism. The results of this study showed no difference in the incidence of thromboembolism (ATE or VTE) between VEGF and EGFR inhibitors, and no difference in the time to thromboembolism, suggesting that the risk of thromboembolism is similar between molecular-targeted therapies. Recently, it has been reported that panitumumab-based chemotherapy is associated with an increased incidence of serious thromboembolism compared to bevacizumab-based chemotherapy [18]. In this study, the overall incidence of thromboembolism was twice as high in the EGFR inhibitor group, but the possibility of a beta error cannot be ruled out due to the limited number of cases.

Khorana et al. [6] reported the following risk factors for thromboembolism in patients with cancer: primary site of cancer type, WBC count > 11,000 cells/μL, platelet count ≥ 350,000 cells/μL, hemoglobin level < 10 g/dL or use of red cell growth factor, and BMI ≥ 35 kg/m^2^. However, BMI ≥ 35 kg/m^2^ is based on the obesity standards in Europe and the United States; therefore, this value is not commonly used in Japan. In this study, we considered the following four items as investigable with reference to previous reports among Japanese populations [7]: WBC count > 11,000 cells/μL, platelet count ≥ 350,000 cells/μL, hemoglobin level < 10 g/dL, and BMI ≥ 25.3 kg/m^2^. The multivariate analysis also showed that more than one risk factor had more influence on thromboembolism than the use of VEGF or EGFR inhibitors. In other words, our results suggest that risk factors for thromboembolism in patients with cancer are more strongly influenced by the cancer itself than by molecular-targeted therapies. However, the fact that it may not be possible to eliminate unknown and unmeasured confounding factors and the small number of events occurring due to the small study size may cause problems with the validity and accuracy of the multivariate analysis.

This study has some limitations. First, it was a small, single-center, retrospective analysis. Second, D-dimer levels were not measured in all patients and could not be included. Similarly, a recent study [19] on colorectal cancer reported that the KRAS status is a risk factor for thromboembolism, but we were unable to evaluate it. Third, the effect of molecular-targeted therapy was not compared with that in the non-use group; therefore, the effect could not be accurately determined. Fourth, all patients with thromboembolism had a CV port in the multivariate analysis and were therefore not included in the statistical analysis.

However, this is one of the few reports directly comparing the incidence of thromboembolism between VEGF and EGFR inhibitors during first-line treatment of unresectable advanced recurrent colorectal cancer in Japanese patients. Additionally, this is the first report to compare the effect of cancer and molecular-targeted drugs on thromboembolism occurrence, showing that the former may have more influence on thromboembolism development.

## Conclusions

Although the sample size of the study was small, the incidence of thromboembolism during first-line treatment of unresectable advanced or recurrent colorectal cancer did not differ between VEGF and EGFR inhibitor use. Additionally, cancer itself may have a greater impact on thromboembolism incidence than the use of molecular-targeted therapies.

## Data Availability

The datasets used and/or analyzed during the current study are available from the corresponding author upon reasonable request.

## Abbreviations

VEGF: Vascular endothelial growth factor
EGFR: Epidermal growth factor receptor
CT: Computed tomography
ATE: Arterial thromboembolism
VTE: Venous thromboembolism
BMI: Body mass index
CV: Central venous
WBC: White blood cell
ROC: Receiver operating characteristic
NO: Nitric oxide
